# Sexual Dimorphism and Age-Related Structural Changes in the Human Larynx: A Morphometric Study with Histological Correlates Relevant to Voice and Diagnostic Assessment

**DOI:** 10.3390/diagnostics16050725

**Published:** 2026-02-28

**Authors:** Alina Anglitoiu, Ahmed Abu-Awwad, Bogdan Anglitoiu, Daniela Gurgus, Daniel Pop, Anca Mihaela Bina, Zoran Laurentiu Popa, Mihai Alexandru Sandesc, Simona-Alina Abu-Awwad

**Affiliations:** 1 Department of ENT, “Victor Babeș” University of Medicine and Pharmacy, 300041 Timisoara, Romania; alina-elisabeta.anglitoiu@umft.ro; 2 Department XV-Discipline of Orthopedics-Traumatology, “Victor Babeș” University of Medicine and Pharmacy, Eftimie Murgu Square, No. 2, 300041 Timisoara, Romania; ahm.abuawwad@umft.ro (A.A.-A.); anglitoiu.bogdan@umft.ro (B.A.); daniel.pop@umft.ro (D.P.); sandesc.mihai@umft.ro (M.A.S.); 3 Research Center University Professor Doctor Teodor Șora, “Victor Babeș” University of Medicine and Pharmacy, Eftimie Murgu Square, No. 2, 300041 Timisoara, Romania; 4 Department of Balneology, Medical Recovery and Rheumatology, Family Discipline, Center for Preventive Medicine, “Victor Babeș” University of Medicine and Pharmacy, 300041 Timisoara, Romania; 5 Department of Obstetrics and Gynecology, “Victor Babeș” University of Medicine and Pharmacy, 300041 Timisoara, Romania; popa.zoran@umft.ro (Z.L.P.); alina.abuawwad@umft.ro (S.-A.A.-A.); 6 Centre for Translational Research and Systems Medicine, “Victor Babeș” University of Medicine and Pharmacy, 300041 Timisoara, Romania; 7 Clinic of Obstetrics and Gynecology, “Pius Brinzeu” County Clinical Emergency Hospital, 300723 Timisoara, Romania

**Keywords:** human larynx, sexual dimorphism, aging, morphometry, cartilage calcification, epiglottic morphology, histology, voice assessment, diagnostic anatomy

## Abstract

**Background/Objectives**: The human larynx exhibits marked sexual dimorphism and undergoes age-related structural remodeling, both of which influence voice characteristics and have important implications for diagnostic assessment. While sex-related differences in laryngeal size are well recognized, the extent to which aging contributes to dimensional versus qualitative structural changes remains incompletely defined. This study aimed to analyze sex- and age-related morphometric and histological characteristics of the human larynx, with a focus on features relevant to voice evaluation and diagnostic interpretation. **Methods**: A cross-sectional anatomical study was conducted on 80 cadaveric human larynges preserved in 10% buffered formalin. Specimens were stratified by sex and age (<30, 30–60, and ≥60 years). Direct morphometric measurements included anteroposterior laryngeal length, thyroid cartilage height, thyroid angle, and relative glottic area. Epiglottic morphology and the presence of laryngeal cartilage calcification/ossification (binary classification: present vs. absent) were recorded. Histological analysis of vocal fold tissue was performed on a stratified subset of specimens. Statistical analysis included *t*-tests, chi-square tests, two-way ANOVA, effect size estimation, and logistic regression. **Results**: Male specimens showed significantly greater anteroposterior length, thyroid cartilage height, and relative glottic area, along with a narrower thyroid angle, compared with females (all *p* < 0.001), with large effect sizes. Age did not significantly influence overall laryngeal dimensions. In contrast, cartilage calcification/ossification increased markedly after the age of 60. Logistic regression identified age ≥ 60 years as the only independent predictor of calcification (OR = 4.37, *p* = 0.039), while sex was not significant. Epiglottic morphology demonstrated a sex-dependent distribution. Histology revealed age-related muscle atrophy and reduced collagen and elastin density. **Conclusions**: Sex defines the baseline morphometric framework of the adult larynx, whereas aging, particularly beyond 60 years, drives qualitative structural degeneration. These findings provide a reproducible anatomical reference for distinguishing sex-related variation from age-related changes in diagnostic assessment.

## 1. Introduction

The human larynx is a highly specialized anatomical structure that plays a central role in both airway protection and voice production, functions that are essential for respiration, communication, and social interaction [1]. Beyond its fundamental anatomical complexity, the larynx is a dynamic organ whose morphology and biomechanical properties change across the human lifespan. These changes are shaped primarily by biological sex and aging and have direct consequences for voice characteristics as well as for clinical evaluation and diagnostic interpretation [2].

Although the general anatomical framework of the larynx has long been established, increasing evidence indicates that subtle morphometric and tissue-level variations related to sex and age can significantly influence functional outcomes. Recent advances in imaging modalities, histological techniques, and acoustic analysis have refined the understanding of how biological sex and aging interact to remodel laryngeal structures, underscoring the importance of detailed anatomical data as a reference for accurate diagnosis [3].

Sexual dimorphism of the larynx becomes particularly evident during puberty, when hormonal influences drive divergent developmental trajectories in males and females [4]. In males, androgen-mediated stimulation results in enlargement of the thyroid cartilage, increased vocal fold length and thickness, and elongation of the vocal tract, changes that produce the characteristic lowering of vocal pitch and deeper timbre [5]. In contrast, females undergo more modest laryngeal growth, with relatively shorter and thinner vocal folds and less pronounced cartilage angulation, features that support higher pitch ranges [6]. These structural differences establish the anatomical substrate of acoustic disparities between male and female voices and provide clinically relevant parameters for voice assessment, imaging interpretation, and forensic applications [4,7].

In addition to sex-related differences, aging exerts a gradual but clinically meaningful influence on the larynx [8]. During early adulthood, laryngeal tissues typically demonstrate optimal elasticity, efficient glottic closure, and stable neuromuscular coordination [9]. With advancing age, a constellation of structural and functional changes, commonly described as presbyphonia, becomes increasingly apparent [10,11]. These changes include ossification and calcification of the laryngeal cartilages, atrophy of intrinsic laryngeal muscles, and alterations of the extracellular matrix characterized by reduced collagen and elastin density. Together, these processes contribute to reduced vocal efficiency, diminished pitch range, breathiness, tremor, and decreased vocal intensity [12].

Importantly, the pattern of age-related laryngeal remodeling differs between sexes. Several studies have shown that older men often experience an elevation of fundamental frequency, whereas older women tend to exhibit a decline, leading to a partial convergence of vocal characteristics in advanced age [13,14,15]. These sex-specific trajectories are relevant for diagnostic evaluation, as they may influence the interpretation of acoustic measurements and structural findings in elderly individuals.

Contemporary research has further highlighted the influence of endocrine and systemic factors on laryngeal structure and function over time. Hormonal fluctuations associated with menopause have been linked to vocal fold edema and thickening, frequently resulting in perceptible voice deepening in women [16]. In men, progressive androgen decline may accentuate vocal fold thinning and vibratory instability [15]. Advances in laryngeal imaging and high-speed videoendoscopy have enabled detailed visualization of these changes, improving the detection of subtle structural alterations relevant to diagnostic practice [17]. At the same time, the integration of anatomical findings with clinical voice science has expanded understanding of how laryngeal aging affects not only professional voice users but also the general aging population, for whom effective communication is closely linked to quality of life [18].

Despite extensive research, gaps remain in correlating anatomical substrates with functional and acoustic outcomes across the lifespan. In particular, the diagnostic implications of sex- and age-related structural variability of the larynx are incompletely defined. The present study aims to analyze sexual dimorphism and age-related structural changes of the human larynx, focusing on morphometric and histological features with relevance for voice evaluation and diagnostic assessment.

## 2. Materials and Methods

### 2.1. Study Design and Specimens

This study was designed as a cross-sectional anatomical and morphometric investigation aimed at assessing the influence of biological sex and aging on the structural characteristics of the human larynx. A total of eighty human larynges were included in the analysis. All specimens were obtained from cadaveric donors and preserved in 10% buffered formalin, a protocol chosen to ensure adequate tissue fixation while preserving anatomical integrity and measurement accuracy.

Specimens were handled in accordance with institutional ethical standards and international regulations governing the use of human anatomical material for research. Only larynges with good macroscopic preservation were considered eligible for inclusion. Specimens showing evidence of traumatic injury, prior cervical or laryngeal surgical intervention, marked pathological alterations, or advanced postmortem degradation were excluded to minimize confounding structural distortion.

Following selection, the larynges were systematically classified according to biological sex (male or female) and stratified into three age categories: under 30 years, between 30 and 60 years, and 60 years or older. This age grouping was chosen to reflect key stages of post-pubertal maturity and age-related structural remodeling. Such stratification allowed for a comparative evaluation of morphometric parameters across both sex and age, facilitating an integrated assessment of developmental and degenerative changes of the laryngeal framework throughout adulthood.

The study was conducted between March 2020 and December 2024 in accordance with the principles of the Declaration of Helsinki and was approved by the Local Ethics Committee of the “Pius Brînzeu” Emergency County Hospital, Timișoara (Decision No. 17/10 February 2020, 10 February 2020).

### 2.2. Morphometric and Histological Assessment

Macroscopic morphometric measurements were performed directly on each specimen under standardized laboratory conditions. High-precision digital calipers with an accuracy of 0.01 mm were used to minimize measurement error and ensure reproducibility. The following anatomical parameters were systematically recorded:

- Anteroposterior (AP) length of the larynx.
- Thyroid cartilage height, measured along the midline.
- Thyroid angle, defined by the intersection of the two thyroid laminae.
- Relative glottic area, calculated as a proportion relative to overall laryngeal dimensions.

**Figure 1:**
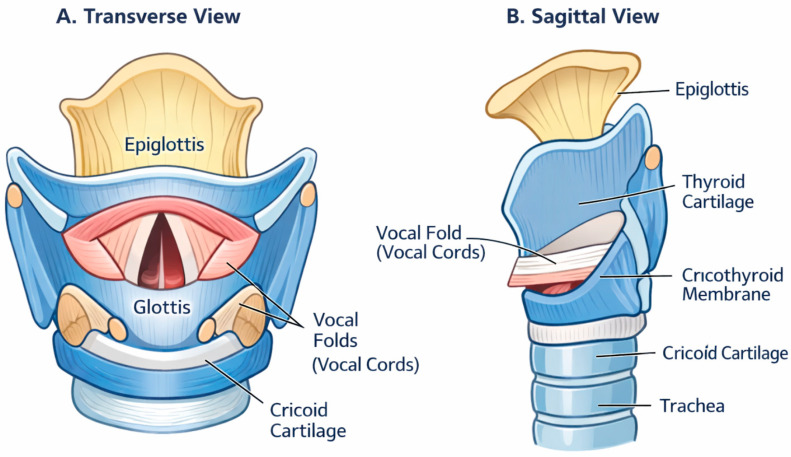
Schematic representation of the human larynx in (**A**) transverse view and (**B**) sagittal view. Major anatomical landmarks are illustrated, including the epiglottis, thyroid cartilage, vocal folds (true vocal cords), glottis, cricoid cartilage, cricothyroid membrane, and trachea. The diagram highlights spatial relationships relevant to morphometric assessment and procedural orientation. Created by the authors for illustrative purposes.

The principal anatomical landmarks and spatial relationships relevant to these measurements are illustrated schematically in Figure 1.

**Figure 2:**
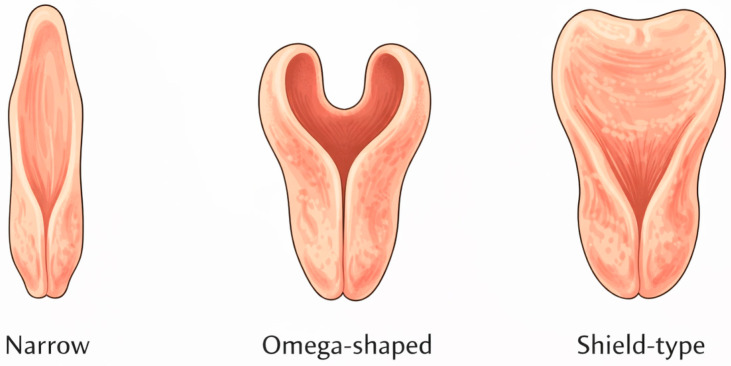
Schematic representation of epiglottis morphological types: narrow, omega-shaped, and shield-type. Created by the authors for illustrative purposes.

In addition to linear measurements, epiglottic morphology was documented and classified as narrow, omega-shaped, or shield-type, according to previously established anatomical criteria [19]. The three epiglottic morphological configurations identified in this study are schematically illustrated in Figure 2.

The presence of laryngeal cartilage calcification/ossification was assessed macroscopically and recorded as a binary variable (present vs. absent). Calcification/ossification was considered present when focal or diffuse whitish, opaque, and visibly rigid areas were identified within the laryngeal cartilage framework, clearly distinguishable from normal elastic or hyaline cartilage.

No semiquantitative grading system was applied. Because macroscopic evaluation does not allow reliable differentiation between true ossification and dystrophic calcification without histochemical or radiologic confirmation, both processes were pooled and analyzed together as a single composite variable (“cartilage calcification/ossification”) for all statistical analyses. Calcification/ossification was considered present when focal or diffuse whitish, opaque, and visibly rigid areas were identified within the cartilage framework, clearly distinguishable from normal elastic or hyaline cartilage tissue.

No semiquantitative grading system was applied. Because macroscopic examination does not reliably allow differentiation between true ossification and dystrophic calcification without histochemical or radiologic confirmation, both processes were pooled and analyzed together as a single composite variable (“cartilage calcification/ossification”) for statistical purposes.

Histological evaluation was performed in a stratified subset of 24 specimens (30% of the total cohort), selected to ensure balanced representation across sex and age categories (4 male and 4 female specimens within each age group: <30, 30–60, and ≥60 years). Within each stratum, specimens were randomly selected among those demonstrating optimal macroscopic preservation and absence of gross pathology.

Tissue samples from the vocal folds were fixed, paraffin-embedded, sectioned at 4–5 µm thickness, and stained with hematoxylin–eosin. Microscopic evaluation was qualitative and descriptive. The analysis focused on muscle fiber morphology, connective tissue density, and the apparent distribution of collagen and elastin fibers. No quantitative morphometric measurements or digital image analysis were performed [4].

### 2.3. Statistical Analysis

Quantitative data were analyzed using standard statistical methods to ensure robust interpretation of morphometric differences. Continuous variables were expressed as mean values with corresponding standard deviations (mean ± SD). Comparisons between male and female specimens, as well as between age groups, were performed using independent-samples *t*-tests for variables with a normal distribution.

Categorical variables, including epiglottic morphology and the presence or absence of cartilage calcification, were analyzed using the chi-square (χ^2^) test. To explore potential interactions between sex and age group, a two-way analysis of variance (ANOVA) was applied, allowing assessment of whether aging-related effects differed between males and females.

Logistic regression analysis was further employed to evaluate whether sex and age independently predicted the occurrence of laryngeal cartilage calcification. Results are reported as odds ratios (ORs) with corresponding 95% confidence intervals (CIs), providing estimates of relative risk within each subgroup.

To complement *p*-values and emphasize the practical relevance of observed differences, effect sizes were calculated using Cohen’s d for continuous variables and Cramer’s V for categorical variables. A *p*-value threshold of <0.05 was considered indicative of statistical significance for all analyses.

## 3. Results

The morphometric and histological evaluation demonstrated clear and consistent patterns of sexual dimorphism, alongside distinct age-related degenerative changes in the human larynx. Structural differences between male and female specimens were evident across multiple parameters, while aging was primarily associated with qualitative tissue changes, particularly cartilage calcification.

Table 1 summarizes the demographic and morphological characteristics of the cadaveric laryngeal specimens included in the analysis. The study cohort showed a balanced distribution of male and female larynges across all predefined age groups, enabling direct comparison of sex- and age-related structural features. The mean age was comparable between sexes, and the wide age range encompassed early adulthood through advanced age, allowing assessment of both post-pubertal anatomy and age-related remodeling. Stratification into groups of <30, 30–60, and ≥60 years of age ensured adequate representation of both mature and degenerative stages of laryngeal structure.

Morphological characteristics, including epiglottic configuration and overall cartilage preservation, were well represented in both sexes. While most baseline demographic and preservation variables were similarly distributed between male and female specimens, distinct sex-specific trends were observed in epiglottic morphology. In contrast, laryngeal cartilage calcification showed a strong association with advancing age rather than sex, being predominantly identified in specimens aged 60 years and older. This distribution is consistent with recognized patterns of age-related structural degeneration of the laryngeal framework.

The overall preservation status of the specimens was predominantly classified as good, supporting the reliability and consistency of the morphometric measurements. Statistical analysis of baseline variables revealed no significant sex-based differences across most demographic and preservation-related parameters (all *p* > 0.05). The sole exception was epiglottic morphology, which demonstrated a statistically significant imbalance between sexes, indicating a sex-dependent distribution pattern. These findings provided a robust and comparable baseline for subsequent morphometric and age-related analyses.

Table 2 presents the sex-based morphometric characteristics of the larynx. Male specimens showed significantly greater anteroposterior length, increased thyroid cartilage height, and a larger relative glottic area compared with female specimens, whereas the thyroid angle was consistently narrower in males. All observed differences reached a high level of statistical significance (*p* < 0.001).

Analysis of laryngeal cartilage calcification/ossification (binary classification: present vs. absent) across age categories demonstrated a low prevalence in specimens younger than 60 years, with comparable frequencies observed in the <30 and 30–60 age groups. All cases of calcification/ossification identified in the cohort (17/80; 21.3%) were confined to the ≥60 years age group, corresponding to 17 of 24 specimens (70.8%) within that subgroup. In contrast, a pronounced increase in calcification was identified in specimens aged 60 years and older, where calcified cartilages were present in more than two-thirds of cases. This age-dependent distribution was statistically significant (χ^2^ = 16.26, df = 2, *p* < 0.001), as detailed in Table 3.

Statistical analysis confirmed robust sex-related differences across all major morphometric parameters of the larynx, with consistently large effect sizes as reflected by Cohen’s d. The strongest effect was observed for the relative glottic area, followed by anteroposterior length and thyroid cartilage height, indicating pronounced structural separation between male and female specimens. All comparisons reached a high level of statistical significance (*p* < 0.001), as detailed in Table 4.

Two-way ANOVA analysis did not reveal a significant main effect of age group on any of the measured laryngeal dimensions. Likewise, no significant interaction between sex and age group was identified, indicating that age-related changes did not differentially affect male and female larynges in terms of overall size. In contrast, biological sex emerged as the primary source of variation in morphometric parameters, confirming that sexual dimorphism represents the dominant determinant of laryngeal dimensions, whereas aging predominantly contributes to qualitative degenerative changes rather than to measurable dimensional variation.

Chi-square analysis further demonstrated a statistically significant association between epiglottic morphology and sex, as well as a stronger relationship between the presence of laryngeal cartilage calcification and age group. Both associations were supported by moderate effect sizes, underscoring their structural relevance. Detailed results of these analyses are presented in Table 5.

Logistic regression analysis identified an age of ≥ 60 years as a significant independent predictor of laryngeal cartilage calcification, with an odds ratio exceeding 4. In contrast, biological sex did not emerge as a significant predictor within the model. These findings indicate that cartilage calcification is primarily driven by aging rather than sex-related structural differences. The complete results of the regression analysis are presented in Table 6.

## 4. Discussion

The present study provides detailed anatomical evidence supporting the presence of marked sexual dimorphism and age-related structural remodeling of the human larynx [4]. The morphometric analysis revealed pronounced and consistent differences between male and female specimens, while evaluation across age groups demonstrated a strong association between advancing age and laryngeal cartilage calcification [11]. Together, these findings contribute to a more refined understanding of how the structural characteristics of the larynx underpin functional voice differences and offer clinically relevant insights for diagnostic assessment, laryngology, and age-related voice evaluation. A broader anatomical context for age-dependent laryngeal changes has been similarly emphasized by Devadas et al. [20].

Our results clearly indicate that male larynges are larger across multiple key dimensions when compared with female specimens. Anteroposterior length, thyroid cartilage height, and relative glottic area were all significantly increased in males, with large effect sizes confirming the robustness and clinical relevance of these differences. Conversely, the thyroid angle was consistently narrower in males, reflecting the well-established anatomical basis of the prominent laryngeal prominence, commonly referred to as the “Adam’s apple.” These sex-specific structural features have direct functional implications: longer and thicker vocal folds in males vibrate at lower fundamental frequencies, resulting in a deeper pitch and timbre, whereas the smaller laryngeal framework and wider thyroid angle observed in females support shorter and thinner vocal folds, which are associated with higher pitch ranges [21].

From a diagnostic perspective, these morphometric differences represent more than descriptive anatomy. They define baseline structural parameters that are essential for accurate interpretation of imaging findings, morphometric measurements, and voice-related assessments. Understanding the expected range of sex-specific laryngeal dimensions is particularly important when distinguishing physiological variation from pathological change, especially in the evaluation of voice disorders or structural abnormalities. To support clinical interpretation, the principal anatomical parameters identified in this study and their corresponding functional vocal implications are summarized in Table 7.

The anatomical differences identified in the present study extend beyond descriptive morphology and carry direct clinical and diagnostic relevance for voice evaluation, airway management, and laryngeal interventions. By integrating detailed morphometric data with established biomechanical principles of phonation, our findings help clarify how sex- and age-related structural characteristics of the larynx translate into functional voice outcomes (Table 7). This structure–function relationship is essential for accurate interpretation of voice-related assessments and imaging findings in routine clinical practice.

From a clinical standpoint, the strong association between advancing age and laryngeal cartilage calcification offers a clear anatomical substrate for presbyphonia. The marked increase in calcification observed after the age of 60 is likely to reduce cartilage elasticity and restrict the fine biomechanical adjustments required for stable and efficient phonation. These changes provide a structural explanation for the reduced vocal range, increased breathiness, vocal instability, and diminished endurance commonly reported in elderly individuals. Recognition of these age-related anatomical constraints is crucial when counseling patients and when designing voice therapy strategies that emphasize functional optimization rather than unrealistic restoration of youthful vocal characteristics.

The observed structural changes also have important implications for airway management, particularly in older patients. Calcified and partially ossified laryngeal cartilages exhibit reduced compliance and increased fragility, making them more susceptible to injury during intubation or surgical manipulation. Awareness of these age-dependent alterations may therefore improve pre-procedural planning and contribute to safer airway management, especially in emergency situations or high-risk clinical settings.

Sex-specific differences in laryngeal framework dimensions remain clinically relevant throughout adulthood and should be considered in surgical planning, including laryngeal framework procedures and gender-affirming voice surgery. The larger laryngeal dimensions and narrower thyroid angle observed in males impose anatomical limits on achievable pitch modification, whereas the smaller laryngeal framework in females supports a different range of therapeutic and surgical outcomes. Establishing realistic goals based on individual structural anatomy is essential for optimizing both functional results and patient satisfaction.

An important finding of the present study is that age-related laryngeal changes do not appear to follow a purely gradual trajectory but become structurally relevant beyond a specific threshold. While overall laryngeal dimensions remained relatively stable across adult age groups, cartilage calcification increased sharply after the age of 60. Logistic regression analysis identified age ≥ 60 years as the sole independent predictor of calcification, suggesting a qualitative shift in tissue properties rather than a progressive change in size. This supports the concept of a structural aging threshold that likely contributes to reduced biomechanical flexibility and age-related voice decline.

Beyond clinical applications, the marked sexual dimorphism observed in key laryngeal parameters also holds relevance for forensic and anthropological contexts. Parameters such as anteroposterior laryngeal length, thyroid cartilage height, and thyroid angle demonstrated large effect sizes, supporting their reliability as anatomical markers for sex estimation. In situations involving fragmented or incomplete skeletal remains, laryngeal cartilage measurements may provide valuable complementary information for biological profiling. The persistence of these sex-related differences across adult age groups further reinforces their forensic utility, despite the superimposed effects of age-related cartilage degeneration.

These findings confirm that sexual dimorphism remains the dominant determinant of adult laryngeal morphology, with direct implications for surgical planning, voice therapy, and forensic sex estimation [22,23].

Epiglottic morphology displayed a distinct sex-related distribution in the present series. The shield-shaped epiglottis was more frequently observed in females, whereas the narrow type predominated in males, while the omega-shaped configuration showed a more balanced distribution between sexes. Although often overlooked in morphometric studies, epiglottic shape may have meaningful implications for both airway physiology and vocal function [24].

Although frequently regarded as a secondary anatomical detail, epiglottic morphology carries several clinically relevant implications [25]. A narrow-type epiglottis, more common in males in our cohort, may complicate airway management and increase the likelihood of difficult intubation, particularly in emergency settings [26,27]. Conversely, configurations such as the shield-type epiglottis, which predominated in females, may influence protective mechanisms against aspiration and alter supraglottic resonance patterns that contribute to vocal quality [28,29]. These observations underscore the importance of considering epiglottic morphology not merely as an anatomical variant but as a factor with practical relevance for anesthesiology, laryngology, and voice care [30].

The age-related patterns of laryngeal cartilage calcification observed in this study are consistent with findings reported in previous anatomical and radiological investigations, supporting the interpretation of cartilage degeneration as a predominantly age-driven process [31,32,33]. Clinically, ossification and calcification of the laryngeal framework have been repeatedly associated with reduced phonatory flexibility; in the present study, these processes were analyzed together as a composite binary variable (calcification/ossification) [34,35,36].

While morphometric analysis constituted the core of the present study, histological evaluation provided complementary insight into the underlying tissue changes. Aging was associated with thinning of intrinsic laryngeal muscles and reduced collagen and elastin density, changes that correlate with cartilage stiffening and declining vocal performance [34,37]. These findings help explain why voice therapy in elderly patients often yields limited results unless combined with strategies targeting respiratory support and resonance optimization [35].

Statistical analyses confirmed the robustness of the observed patterns. High t values and large effect sizes demonstrated that sex-related morphometric differences were both statistically and clinically significant [38,39]. Chi-square testing revealed meaningful associations between epiglottic morphology and sex, as well as between cartilage calcification and age, supporting the biological relevance of these structural relationships [40]. Two-way ANOVA identified no interaction between sex and age group, indicating that aging affects male and female larynges in a broadly similar manner, with age-related vocal decline shaped primarily by structural differences established earlier in life [41].

Taken together, the morphometric and age-related findings provide a clear anatomical framework for understanding well-established differences in voice pitch, timbre, and stability across the lifespan. Men, characterized by larger laryngeal dimensions, typically exhibit lower pitch, while aging-related vocal fold thinning and loss of elasticity may lead to pitch elevation in later life [2,15]. Women, who begin with a higher pitch, often experience a postmenopausal decline related to hormonal changes and vocal fold thickening. These opposing trajectories result in a partial convergence of vocal characteristics with advancing age, consistent with the patterns observed in the present study [13,15].

From a clinical perspective, these findings highlight the importance of counseling voice professionals and patients regarding anatomical and age-related limitations. Early identification of presbyphonia should guide rehabilitation toward functional optimization rather than attempts to restore youthful voice qualities [42]. Surgeons must likewise account for cartilage variability and ossification, factors that directly influence procedural complexity and surgical outcomes [43].

A multidisciplinary approach remains essential, involving otolaryngologists, speech therapists, endocrinologists, and geriatricians. Sexual dimorphism defines the baseline laryngeal anatomy, while aging progressively alters cartilage and muscle integrity, jointly shaping diagnostic evaluation and therapeutic strategies. In forensic practice, morphometric parameters such as anteroposterior length, thyroid angle, and cartilage height continue to provide reliable indicators of sex, supported by strong effect sizes [44,45].

Overall, the present findings confirm that sexual dimorphism establishes the structural framework of the human larynx, while aging superimposes degenerative changes, particularly cartilage calcification and ossification. The identification of age ≥ 60 years as an independent predictor of calcification underscores the universality of this process, positioning sex and age as complementary structural determinants of voice evolution across the human lifespan [40,43].

Interpreting our procedural mapping also requires acknowledging well-described inter-individual variation in laryngeal anatomy. Differences in overall laryngeal size and configuration (including sex-related morphology) and age-related tissue remodeling can modify surface landmarks, membrane compliance, and spatial relationships relevant to percutaneous access in cadaveric specimens. In our series, although we did not perform a formal statistical, stratified analysis of procedural parameters by sex or age, we noted consistent descriptive patterns during mapping: male larynges generally required deeper needle advancement to reach the target region, in line with their larger anteroposterior dimensions and thyroid cartilage height. In older specimens (particularly ≥60 years), increased cartilage rigidity and reduced tissue compliance were more frequently encountered during membrane traversal, plausibly reflecting calcification/ossification-related changes. These observations support the concept that percutaneous true vocal fold access benefits from individualized trajectory planning rather than a “one-size-fits-all” approach, consistent with recent work reporting needle insertion angles for thyrohyoid and cricothyroid membrane access [46]. Future studies should integrate morphometric measurements with standardized procedural metrics (angle, depth, and resistance) and apply stratified statistical analyses across sex and age groups to improve generalizability and procedural precision.

### Strengths, Limitations, and Future Directions

A major strength of the present study is the relatively large number of cadaveric larynges analyzed (*n* = 80), systematically stratified by sex and age group. This design enabled robust comparative analyses and reduced variability related to external confounding factors. The use of direct anatomical measurements obtained with high-precision instruments ensured objective and reproducible morphometric data, particularly for key parameters such as anteroposterior laryngeal length, thyroid cartilage height, thyroid angle, and relative glottic area. An additional strength lies in the integration of macroscopic morphometry with histological evaluation, which provides a comprehensive view of both structural and tissue-level changes associated with aging. Finally, the application of rigorous statistical methods, including effect size estimation (Cohen’s d and Cramer’s V) and logistic regression modeling, strengthened the interpretation of results by emphasizing not only statistical significance but also structural and potential clinical relevance.

Several limitations should nevertheless be acknowledged. Although clear morphometric differences between male and female larynges were confirmed, many of these parameters, such as anteroposterior length, thyroid angle, and cartilage height, have been previously described in the literature and may therefore be regarded as confirmatory rather than novel. The added value of the present study lies primarily in two areas: first, the systematic evaluation of epiglottic morphology according to sex, a feature that is less frequently addressed and may have meaningful implications for airway assessment; and second, the use of logistic regression analysis to demonstrate that age ≥ 60 years represents an independent predictor of laryngeal cartilage calcification, providing quantitative support for a threshold-related aging effect. Emphasizing these aspects is important to distinguish the present work from purely descriptive anatomical studies. The procedural observations related to percutaneous access were descriptive and were not supported by inferential statistics or a priori stratified modeling by sex and age. Accordingly, these findings should be interpreted as hypothesis-generating and warrant confirmation in larger, prospectively designed studies.

As an anatomical investigation based on cadaveric material, direct correlations with functional voice outcomes or clinical acoustic data could not be established. Detailed information regarding donors’ medical history, hormonal status, or environmental exposures was unavailable, limiting causal inference regarding the mechanisms underlying the observed structural changes. Postmortem alterations and variability in tissue preservation may have influenced certain measurements, particularly in specimens with advanced ossification or cartilage desiccation. In addition, the broad age stratification (<30, 30–60, ≥60 years) does not fully capture biologically critical transitions such as puberty or menopause. The fact that all specimens originated from a single regional population may further limit generalizability to other ethnic or geographic groups.

Future research should aim to integrate detailed anatomical data with functional assessments, including acoustic voice analysis and dynamic laryngoscopic or imaging-based evaluation. Expanding analyses to more diverse populations would improve understanding of genetic, cultural, and environmental influences on sexual dimorphism and age-related laryngeal remodeling. A more refined age stratification could help delineate transitional phases of laryngeal development and degeneration. Further investigation of hormonal influences across the lifespan, including puberty, adulthood, and postmenopause, would clarify endocrine contributions to structural and functional voice changes. Advances in radiologic imaging and three-dimensional reconstruction techniques may enhance both clinical and forensic applications. Ultimately, longitudinal studies following individuals over time would provide decisive insight into the dynamic evolution of the larynx and support the development of preventive, diagnostic, and rehabilitative strategies for age-related voice decline.

## 5. Conclusions

This morphometric and histological study demonstrates that sexual dimorphism and aging exert distinct effects on the structural characteristics of the human larynx. Biological sex emerged as the primary determinant of laryngeal size and geometry, with male specimens showing significantly greater anteroposterior length, thyroid cartilage height, and relative glottic area, as well as a narrower thyroid angle, all with large effect sizes. In contrast, aging did not significantly influence overall laryngeal dimensions but was strongly associated with qualitative structural remodeling. Cartilage calcification/ossification increased sharply after the age of 60, and logistic regression identified age ≥ 60 years as the only independent predictor of calcification, indicating a threshold-related degenerative process rather than progressive dimensional change. Epiglottic morphology showed a sex-dependent distribution, highlighting an additional structural feature relevant for airway evaluation. Histological findings of muscle atrophy and reduced collagen and elastin density further supported the observed age-related stiffening of the laryngeal framework. Together, these results provide a clear anatomical reference for distinguishing sex-related baseline anatomy from age-related degenerative changes in diagnostic assessment.

## Figures and Tables

**Table 1:** Demographic and morphological characteristics of cadaveric human larynges stratified by sex.

Variable		Total (*n* = 80)	Females (*n* = 38)	Males (*n* = 42)	*p* Value (Male vs. Female)
Age (mean ± SD)		52.8 ± 18.4	53.9 ± 17.6	51.8 ± 19.2	0.81
Age range		18–89	19–85	18–89	-
Age group	<30 yrs	22 (27.5%)	10 (26.3%)	12 (28.6%)	0.82
	30–60 yrs	34 (42.5%)	16 (42.1%)	18 (42.8%)	0.94
	≥60 yrs	24 (30.0%)	12 (31.5%)	12 (28.6%)	0.76
Cartilage calcification/ossification present (overall cohort)		17 (21.3%)	8 (21.0%)	9 (21.4%)	0.96
Epiglottis type	Narrow	36 (45.0%)	12 (31.5%)	24 (57.1%)	0.02
	Omega	14 (17.5%)	6 (15.7%)	8 (19.0%)	<0.01
	Shield	30 (37.5%)	20 (52.6%)	10 (23.8%)	<0.01
Preservation	Good	61 (76.3%)	30 (78.9%)	31 (73.8%)	0.59
	Moderate	17 (21.3%)	7 (18.4%)	10 (23.8%)	0.55
	Poor	2 (2.5%)	1 (2.6%)	1 (2.3%)	0.94

Data are reported as absolute values, with percentages shown in parentheses. Percentages in the “Total” column are calculated relative to the entire study sample (*n* = 80). Percentages in the “Females” and “Males” columns are calculated relative to the total number of female (*n* = 38) and male (*n* = 42) specimens, respectively. Age is expressed as mean ± standard deviation (SD). “Cartilage calcification/ossification” denotes the presence of macroscopic cartilage calcification/ossification within the entire cohort (17/80). All cases were observed in the ≥60 years subgroup (17/24 specimens within that category). “Preservation” reflects the overall macroscopic condition of the laryngeal specimens at the time of assessment.

**Table 2:** Sex-based morphometric measurements of the human larynx.

Variable	Female	Male	*p* Value
AP Length (mm)	36.4 ± 2.57	44.98 ± 2.98	<0.001
Thyroid height (mm)	22.6 ± 2.54	30.27 ± 2.73	<0.001
Glottic area (rel.)	1.0 ± 0.05	1.27 ± 0.08	<0.001
Thyroid angle (°)	98.56 ± 5.77	70.26 ± 3.85	<0.001

Data are presented as mean ± standard deviation (SD). Comparisons between female and male specimens were performed using independent-sample *t*-tests. *p* values reflect the statistical significance of sex-based differences for each morphometric parameter. AP, anteroposterior; rel., relative.

**Table 3:** Age-group distribution of laryngeal cartilage calcification/ossification (present vs. absent).

Age Group	Present	Absent
<30	5 (16.66%)	17 (34.00%)
30–60	8 (26.66%)	26 (52.00%)
≥60	17 (56.66%)	7 (14.00%)

Values are counts with percentages in parentheses. Calcification was significantly more frequent in specimens aged ≥ 60 years (*p* < 0.001). Overall comparison across age groups: χ^2^ = 16.26, df = 2, *p* < 0.001.

**Table 4:** Sex-based comparative statistical analysis of laryngeal morphometric parameters.

Index	*t*	*p*	Cohen_d
AP length (mm)	13.82	<0.001	3.07
Thyroid height (mm)	13.0	<0.001	2.9
Glottic area (rel.)	17.32	<0.001	3.79

Results of independent-sample *t*-tests comparing morphometric laryngeal parameters between male and female specimens. Effect sizes are reported as Cohen’s d, indicating the magnitude of sex-related differences. *p* values reflect the level of statistical significance for each comparison. AP, anteroposterior; rel., relative.

**Table 5:** Chi-square analysis of associations between epiglottic morphology, cartilage calcification, sex, and age group.

Index	Statistic	df	*p*-Value	Cramers_V
0 (Chi-square: Epiglottis Type ~ Sex)	7.44	2	<0.05	0.3
0 (Chi-square: Calcification/Ossification ~ Age Group)	16.26	2	<0.001	0.45

Results of chi-square (χ^2^) tests evaluating the associations between epiglottic morphology and biological sex and between the presence of laryngeal cartilage calcification and age group. Effect sizes are reported as Cramer’s V, reflecting the strength of association. Degrees of freedom (df) and corresponding *p* values are provided for each comparison.

**Table 6:** Logistic regression analysis of factors associated with laryngeal cartilage calcification/ossification.

Index	OR	2.5%	97.5%	*p*-Value
Intercept	0.56	0.19	1.66	0.292
Age ≥ 60	4.37	1.07	17.79	0.039
Male sex	0.42	0.11	1.61	0.208

Results of logistic regression analysis evaluating the association between age, sex, and the presence of laryngeal cartilage calcification. Data are reported as odds ratios (ORs) with corresponding 95% confidence intervals (CIs). *p* values indicate the statistical significance of each predictor. Age of ≥ 60 years was identified as a significant independent predictor of cartilage calcification, whereas sex was not statistically significant.

**Table 7:** Anatomical laryngeal parameters and their functional vocal implications.

Anatomical Parameter	Sex-/Age-Related Pattern Observed in This Study	Functional Vocal Implication	Clinical Relevance
Anteroposterior (AP) length of the larynx	Significantly greater in males across all age groups (*p* < 0.001); no significant age-related variation	Increased vocal fold length is associated with lower fundamental frequency (F0) and deeper vocal timbre	Explains lower pitch in male voices; relevant for voice therapy and surgical planning
Thyroid cartilage height	Significantly higher in males than females (*p* < 0.001); stable across age groups	Larger laryngeal framework supports greater vocal fold mass and lower vibratory frequency	Important for gender-affirming voice surgery and realistic pitch expectations
Thyroid angle	Markedly narrower in males compared to females (*p* < 0.001); not influenced by age	Narrower angle increases vocal fold tension and projection, contributing to voice strength and clarity	Relevant for laryngeal framework surgery and forensic sex estimation
Relative glottic area	Significantly larger in males (*p* < 0.001); no significant age effect	Larger glottic area allows higher sound pressure levels and increased vocal intensity	Relevant for professional voice users and vocal endurance
Epiglottis morphology	Sex-dependent distribution: narrow type more frequent in males, shield type more common in females (*p* < 0.05)	Epiglottic shape may influence supraglottic resonance and airway protection mechanisms	Potential implications for airway management and subtle voice quality differences
Laryngeal cartilage calcification	Strongly associated with age ≥ 60 years (OR = 4.37, *p* = 0.039); no independent sex effect	Reduced cartilage elasticity limits fine biomechanical control of phonation	Key structural contributor to presbyphonia and age-related voice instability
Intrinsic laryngeal muscle integrity (histology)	Age-related muscle fiber atrophy and reduced collagen/elastin density	Decreased vibratory efficiency and increased breathiness in aging voice	Supports combined rehabilitative approaches in elderly voice therapy

## Data Availability

The data presented in this study are available upon request from the corresponding author. The data are not publicly available due to hospital policy.
